# Clinical Impact of HLA‐DPB1 Permissiveness Models on Acute GVHD and Survival After Unrelated Allogeneic HCT: A Single‐Centre Brazilian Cohort

**DOI:** 10.1111/tan.71064

**Published:** 2026-11-04

**Authors:** Matilde Romero, Vinicius Navega Stelet, Mirian Carvalho de Souza, Anna Paula Villela, Gabriela Lemos, Renata Binato, Eliana Abdelhay

**Affiliations:** ^1^ Immunogenetics Laboratory, Specialized Laboratories Division National Cancer Institute Rio de Janeiro Brazil; ^2^ Health Technology Assessment Division National Cancer Institute Rio de Janeiro Brazil

**Keywords:** acute graft‐versus‐host disease, allogeneic haematopoietic stem cell transplantation, HLA‐DPB1, immunogenetics, PIRCHE, T‐cell epitope

## Abstract

HLA‐DPB1 mismatches are frequent in unrelated allogeneic haematopoietic stem cell transplantation (allo‐HCT), even among donor–recipient pairs matched at conventional HLA loci. Models including T‐cell epitope (TCE), expression and Predicted Indirectly Recognisable HLA Epitopes (PIRCHE) have been proposed to assess HLA‐DPB1 permissiveness. To evaluate the impact of HLA‐DPB1 permissiveness assessed by TCE, expression and PIRCHE models on acute graft‐versus‐host disease (aGVHD) and overall survival after unrelated donor allo‐HCT. This retrospective cohort study included 137 recipients who underwent unrelated allo‐HCT between 2010 and 2024 at the Bone Marrow Transplant Centre of the Brazilian National Cancer Institute (CEMO‐INCA). High‐resolution HLA‐DPB1 genotyping was performed by next‐generation sequencing. HLA‐DPB1 permissiveness was evaluated using TCE classification, rs9277534 expression‐associated polymorphism and PIRCHE‐I/II scores. Multivariable analysis for Grade II–IV aGVHD were performed using the Fine and Grey subdistribution hazards model, and overall survival was assessed using Kaplan–Meier and Cox models. Among 85 donor–recipient pairs initially matched at HLA 10/10, only 20 (23.5%) were also matched at HLA‐DPB1. Double HLA‐DPB1 GVH‐direction mismatches suggested an increased risk of Grade II–IV aGVHD (sHR = 2.82; *p* = 0.036), as did TCE‐1 mismatches (sHR = 3.27; *p* = 0.041) and the highest PIRCHE‐I score tertile (sHR = 3.36; *p* = 0.052) suggested a borderline association. In HLA 9/10 transplants, a single high‐expression GVH‐direction mismatch (sHR = 3.33; *p* = 0.025), as did TCE‐1 mismatches (sHR = 3.64; *p* = 0.015) and the highest PIRCHE‐I (sHR = 7.02; *p* = 0.003), with a similar trend for PIRCHE‐II scores tertile suggested association with increased aGVHD risk. None of the HLA‐DPB1 permissiveness models or PIRCHE were associated with overall survival. TCE‐ and PIRCHE‐based HLA‐DPB1 permissiveness models suggested association with aGVHD risk after unrelated allo‐HCT, but given the small size of these subgroups, these findings should be considered hypothesis‐generating.

## 1 Introduction

Allogeneic haematopoietic stem cell transplantation (allo‐HCT) remains one of the most effective curative therapies for hematologic malignancies. However, successful transplantation critically depends on donor–recipient compatibility within the HLA system. Because only a minority of patients have a matched sibling donor (MSD), the identification of unrelated donors (MUD) through the Brazilian National Registry of Voluntary Bone Marrow Donors (REDOME) and international registries has become essential to expand access to transplantation. Nevertheless, even when donor–recipient compatibility is achieved at the major HLA loci (HLA‐A, ‐B, ‐C, ‐DRB1 and ‐DQB1; 10/10 matched transplantation), mismatches at the HLA‐DPB1 locus are frequently observed. Furthermore, a fully matched donor is not always available, making 9/10 HLA‐matched unrelated donors an important alternative for allo‐HCT [1, 2].

HLA‐DPB1 mismatches have consistently been associated with an increased risk of acute graft‐versus‐host disease (aGVHD) after unrelated donor HCT, regardless of compatibility at other HLA Class I and Class II loci. Conversely, these mismatches may also enhance graft‐versus‐leukaemia (GVL) effects, contributing to lower relapse rates [3, 4]. Subsequent studies demonstrated that not all HLA‐DPB1 mismatches exert the same clinical impact, proposing that some mismatches are clinically permissive, eliciting weaker alloimmune responses, whereas others are non‐permissive and associated with poorer transplant outcomes [5, 6, 7, 8, 9].

Three major models have been proposed to evaluate HLA‐DPB1 permissiveness, each grounded in a distinct immunological principle. The T‐cell epitope (TCE) model classifies HLA‐DPB1 alleles according to their capacity to elicit alloreactive T‐cell responses, distinguishing permissive mismatches, those sharing the same immunogenicity group between donor and recipient from non‐permissive ones [10, 11]. The expression model is based on an exon 3 sequence motif in strong linkage disequilibrium with the rs9277534 polymorphism, located in the 3′ untranslated region; this variant modulates HLA‐DPB1 surface expression on antigen‐presenting cells, with mismatches involving high‐expression alleles thought to elicit stronger alloimmune activation than those involving low‐expression alleles [12, 13, 14]. The Predicted Indirectly Recognisable HLA Epitopes (PIRCHE) model, in turn, estimates the number of indirectly recognisable epitopes generated from donor‐recipient HLA mismatches, based on the principle that allogeneic HLA peptides can be processed and presented by shared HLA molecules, thereby triggering indirect T‐cell allorecognition [15, 16].

Given the limited number of studies evaluating the combined impact of HLA‐DPB1 permissiveness models and PIRCHE scores on transplant outcomes, the present study aimed to evaluate HLA‐DPB1 permissiveness using the TCE, expression and PIRCHE models in the context of aGVHD and overall survival following unrelated donor allo‐HCT. Analyses were performed in both fully matched (10/10) and mismatched (9/10) transplant settings. In addition, this study sought to assess the utility of these models as risk‐stratification strategies to refine donor selection and potentially reduce post‐transplant complications.

## 2 Methods

### 2.1 Study Design and Population

This retrospective cohort study included recipients who underwent unrelated allogeneic haematopoietic stem cell transplantation (allo‐HCT) for haematologic malignancies between 2010 and 2024 at the Bone Marrow Transplant Centre (CEMO) of the Brazilian National Cancer Institute (INCA), Rio de Janeiro, Brazil, together with their respective donors.

Eligibility criteria included: (i) unrelated allo‐HCT performed between 2010 and 2024; (ii) use of bone marrow or peripheral blood stem cells as the graft source; and (iii) availability of complete clinical follow‐up data in medical records and institutional databases. Both adult and paediatric recipients were included.

Clinical and demographic variables collected included recipient and donor age, sex, underlying disease, disease risk, recipient CMV serostatus, conditioning regimen, stem cell source, graft‐versus‐host disease prophylaxis, HLA compatibility, incidence of aGVHD and survival outcomes.

### 2.2 Acute GVHD Classification

Acute GVHD was classified according to the National Institutes of Health (NIH) consensus criteria established in 2005 [17]. Recipients were categorised according to aGVHD severity as Grades 0–I or Grades II–IV for statistical analyses.

### 2.3 HLA Genotyping and HLA‐DPB1 Permissiveness Analysis

High‐resolution HLA‐DPB1 genotyping was performed using next‐generation sequencing (NGS) by hybrid capture methodology. Sequencing data generated on the MiSeq platform (Illumina, San Diego, CA, USA) were analysed using AlloSeq Assign software version 1.0.6 (CareDx, Brisbane, CA, USA).

HLA‐DPB1 mismatches were categorised as permissive or non‐permissive according to version 3.65.0 of the T‐cell epitope (TCE) algorithm provided by the IPD‐IMGT/HLA database. Permissiveness was assessed directionally on the difference between the patient's and donor's TCE (TCE1, TCE2 and TCE3) groups.

HLA‐DPB1 expression levels were classified as high‐ or low‐expression based on the exon 3 sequence motif in strong linkage disequilibrium with the rs9277534 polymorphism, located in the 3′untranslated region. Alleles associated with the rs9277534A variant were classified as low‐expression alleles, whereas alleles associated with rs9277534G were classified as high‐expression alleles.

PIRCHE‐I and PIRCHE‐II scores were calculated to estimate the predicted number of indirectly recognisable HLA epitopes generated from donor–recipient mismatches using version 4.2.77 on the TxPredictor platform (https://www.pirche.com/pirche/#/) for all donor‐recipient pairs (10/10 and 9/10 matches).

### 2.4 Statistical Analysis

Clinical outcomes analysed included OS and incidence of Grade II–IV aGVHD. Descriptive statistics were used to summarise patient, donor and transplant characteristics. Continuous variables were expressed as medians and ranges, and comparisons between groups were performed using the Wilcoxon rank‐sum test. Categorical variables were expressed as frequencies and percentages and compared using the Chi‐square test or Fisher's exact test, as appropriate.

To address the occurrence of early mortality before aGVHD onset, the cumulative incidence of Grade II–IV aGVHD was estimated using a competing risks framework, where death without aGVHD was treated as the competing event. Recipients alive without Grade II–IV aGVHD were censored at the last clinical follow‐up. Cumulative incidence was estimated with the Aalen–Johansen estimator, reported at 24 months after transplantation and compared across groups with Grey's test. Multivariable analysis for Grade II–IV aGVHD was performed using the Fine and Grey subdistribution hazards model, and the clinical impact of HLA‐DPB1 permissiveness was reported as subdistribution hazard ratios (sHR) with 95% confidence intervals (CI).

Overall survival (OS) was calculated from the date of transplantation until death from any cause or the last clinical follow‐up. OS probabilities were estimated using the Kaplan–Meier method, and survival curves were compared using the log‐rank test. Multivariable analysis for OS was conducted using Cox proportional hazards regression models to estimate hazard ratios (HR) and 95% CIs.

To account for potential confounding factors across the 2010–2024 transplant era, candidate covariates (transplant period, underlying disease risk, conditioning intensity, stem cell source, recipient CMV serostatus and GVHD prophylaxis) were screened univariably in the 104 pairs with complete HLA‐DPB1 typing, and those with *p* ≤ 0.20 were considered for inclusion in the multivariable models. Given the limited number of Grade II–IV aGVHD events, the number of covariates in the multivariable Fine–Grey models was restricted to maintain an adequate events‐per‐variable ratio [18]. Because post‐transplant relapse dates were not systematically recorded, death attributed to relapse was used as a proxy for relapse. Relapses‐related mortality was compared between groups using Fisher's exact test, both in the whole cohort and among deaths with known causes.

The proportional hazards assumption for the Cox models were evaluated using Schoenfeld residuals. Missing data were handled by complete‐case analysis, with the number of missing values reported for each variable. All statistical analyses were performed using R software (version 4.4.3), and two‐sided *p* values ≤ 0.05 were considered statistically significant.

## 3 Results

### 3.1 Characteristics of the Allo‐HCT Cohort According to HLA Match

A total of 137 recipients who underwent unrelated allo‐HCT were included in this study. Baseline characteristics according to HLA match are summarised in Table 1. The groups HLA 10/10 and HLA 9/10 were comparable for all recipient, donor and transplant characteristics, except for transplant period: HLA 9/10 transplants were more frequent in 2010–2017 than in 2018–2024 (67.7% vs. 32.3% of each group performed in the earlier period; *p* = 0.042).

**TABLE 1:** Clinical and demographic characteristics of donor–recipient pairs who underwent unrelated allogeneic haematopoietic stem cell transplantation (allo‐HCT) between 2010 and 2024 according to HLA match.

Characteristics of the cohort according to HLA match	allo‐HCT—HLA match				Overall		*p*
	10/10		9/10				
	*N*	%	*N*	%	*N*	%	
Overall	106	77.4	31	22.6	137	100.0	
Recipient Characteristics							
Age group, years							
Median (range)	22 (2–68)		17 (4–59)				
≤ 15	37	34.9	14	45.2	51	37.2	
16–25	22	20.8	3	9.7	25	18.2	0.783^a^
26–35	14	13.2	3	9.7	17	12.4	
36–45	13	12.3	4	12.9	17	12.4	
≥ 46	20	18.9	7	22.6	27	19.7	
Gender							
Male	70	66.0	21	67.7	91	66.4	> 0.999^b^
Female	36	34.0	10	32.3	46	33.6	
Disease at HCT							
ALL	51	48.1	14	45.2	65	47.4	0.807^b^
AML	26	24.5	10	32.3	36	26.3	
CML	15	14.2	4	12.9	19	13.9	
MDS	12	11.3	2	6.5	14	10.2	
NHL	2	1.9	1	3.2	3	2.2	
Disease risk							
high/very high	50	47.2	16	51.6	66	48.2	0.688^b^
intermediate/low	56	52.8	15	48.4	71	51.8	
CMV serostatus							
IgG−	24	22.6	8	25.8	32	23.4	0.810^b^
IgG+	82	77.4	23	74.2	105	76.6	
Donor characteristics							
Age group							
Median (range)	35 (20–56)		32 (21–48)				
≤ 25	8	7.5	4	12.9	12	8.8	0.214^a^
26–35	37	34.9	10	32.3	47	34.3	
36–45	30	28.3	5	16.1	35	25.5	
≥ 46	12	11.3	3	9.7	15	10.9	
Missing	19	17.9	9	29.0	28	20.4	
Gender							
Male	66	62.3	20	64.5	86	62.8	> 0.999^b^
Female	40	37.7	11	35.5	51	37.2	
Transplant characteristics							
HCT Period							
2010–2017	49	46.2	21	67.7	70	51.1	0.042^b^
2018–2024	57	53.8	10	32.3	67	48.9	
Donor/recipient gender match							
Male/Male	47	44.3	15	48.4	62	45.3	0.990^b^
Male/Female	19	17.9	5	16.1	24	17.5	
Female/Male	23	21.7	6	19.4	29	21.2	
Female/Female	17	16.0	5	16.1	22	16.1	
Graft type							
Bone marrow	73	68.9	24	77.4	97	70.8	0.501^b^
Peripheral blood	33	31.1	7	22.6	40	29.2	
Conditioning regimen							
Myeloablative	93	87.7	29	93.5	122	89.1	0.530^b^
Non‐myeloablative	6	5.7	0	0.0	6	4.4	
Reduced intensity	7	6.6	2	6.5	9	6.6	
GvHD prophylaxis							
CsA + MTX	94	88.7	27	87.1	121	88.3	0.090^b^
FK506 + MTX	12	11.3	4	12.9	16	11.7	
Degree aGVHD incidence^c^							
no aGVHD	58	54.7	14	29.8	72	52.6	
I	14	13.2	2	4.3	16	11.7	
II	25	23.6	11	23.4	36	26.3	
III	7	6.6	3	6.4	10	7.3	
IV	2	1.9	1	2.1	3	2.2	
aGVHD group^c^							
0–I	72	67.9	16	51.6	88	64.2	
II–IV	34	32.1	15	48.4	49	35.8	

Abbreviations: aGVHD, acute graft‐versus‐host disease; HCT, haematopoietic cell transplantation.

^a^ Wilcoxon rank‐sum (Mann–Whitney U) test.

^b^ Fisher's exact test (used throughout instead of chi‐square due to small expected cell counts in several categories). Bold *p* values indicate *p* < 0.05.

^c^ aGVHD (descriptive; formal comparison by Fine‐Grey/Grey test, not Fisher—see main text).

In both groups, most donor–recipient pairs were male. Paediatric recipients younger than 15 years represented the largest age subgroup in both HLA 10/10 (34.9%) and HLA 9/10 (45.2%) transplants. Most donors were between 26 and 35 years of age. Nearly half of the recipients had high or very high disease risk (48.2%), and the majority were CMV‐seropositive. Acute lymphoblastic leukaemia (ALL) was the predominant underlying disease, and bone marrow was the main stem cell source in both transplant groups. Myeloablative conditioning regimens were more frequently used, and all recipients received prophylaxis based on calcineurin inhibitors (CNI); the combination of cyclosporine and methotrexate was the most frequently administered regimen for aGVHD. The incidence of Grade II–IV aGVHD was 32.1% among recipients who underwent HLA 10/10 transplantation and 48.4% among those who underwent HLA 9/10 transplantation.

### 3.2 Cumulative Incidence of Grade II–IV aGVHD

Cumulative incidence of Grade II–IV aGVHD in the first 24 months after HCT was performed comparing patients with 10/10 matched (*n* = 106) versus 9/10 mismatched (*n* = 31) transplants in the overall cohort. The 10/10 group showed a cumulative incidence of 32.3% (95% CI: 23.4%–41.2%), while the 9/10 group showed 48.4% (95% CI: 30.6%–66.2%), both reaching a plateau by approximately 4–5 months. Although the numerical difference suggests higher risk in the mismatched group, Grey's test did not reach statistical significance (*p* = 0.110) (Figure 1). The cumulative incidence of death without prior Grade II–IV aGVHD did not differ significantly between groups (*p* = 0.926).

**FIGURE 1:**
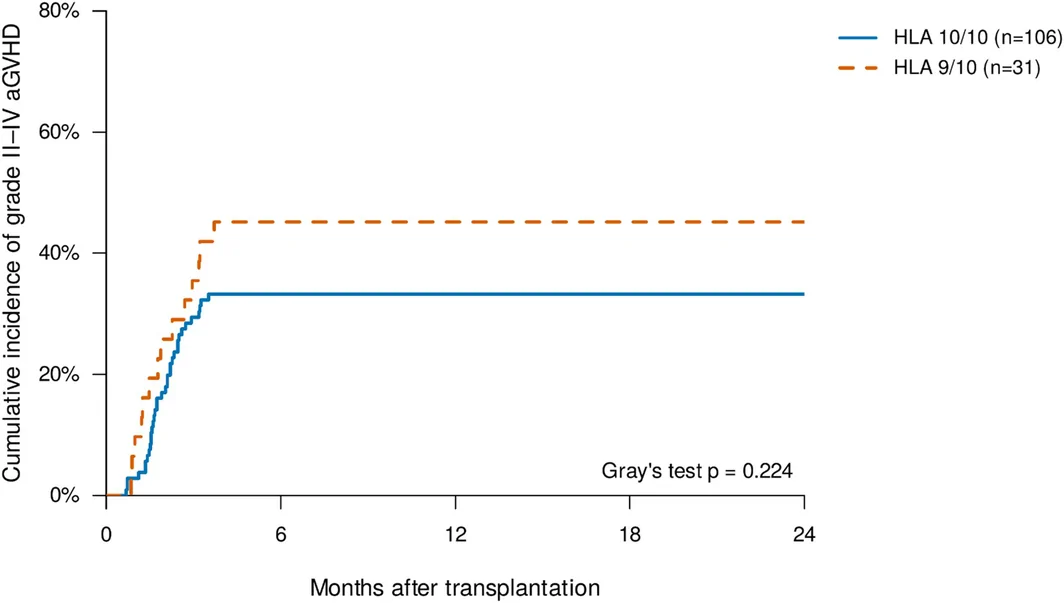
Cumulative incidence of Grade II–IV aGVHD by classical HLA match status.

Among the 104 donor–recipient pairs with complete HLA‐DPB1 genotyping, 85 were initially HLA 10/10 matched, while 19 were HLA 9/10 matched. The cumulative incidence of Grade II–IV aGVHD within the first 24 months after HCT was analysed according to HLA matching status and the number of HLA‐DPB1 mismatches, resulting in five groups: 12/12 match (*n* = 20), 10/10 + 1 DPB1 mismatch (*n* = 47), 10/10 + 2 DPB1 mismatches (*n* = 18), 9/10 + 1 DPB1 mismatch (*n* = 13) and 9/10 + 2 DPB1 mismatches (*n* = 6). A risk gradient is observed across groups: the lowest 24‐month cumulative incidence occurred in the 12/12 match group (20.3%), followed by 10/10 + 1MM DPB1 (25.5%). The 10/10 + 2MM DPB1 and 9/10 + 1MM DPB1 groups showed the highest incidences, 55.6% and 69.2% respectively with latter reaching a plateau by approximately 3–4 months. The 9/10 + 2MM DPB1 group (*n* = 6) showed an apparently lower incidence (16.7%), but this should be interpreted with caution given the very small sample size, yielding an unstable estimate.

The global Grey's test across the five groups was statistically significant (*p* = 0.004), indicating a difference in cumulative incidence of Grade II–IV aGVHD across these groups (Figure 2).

**FIGURE 2:**
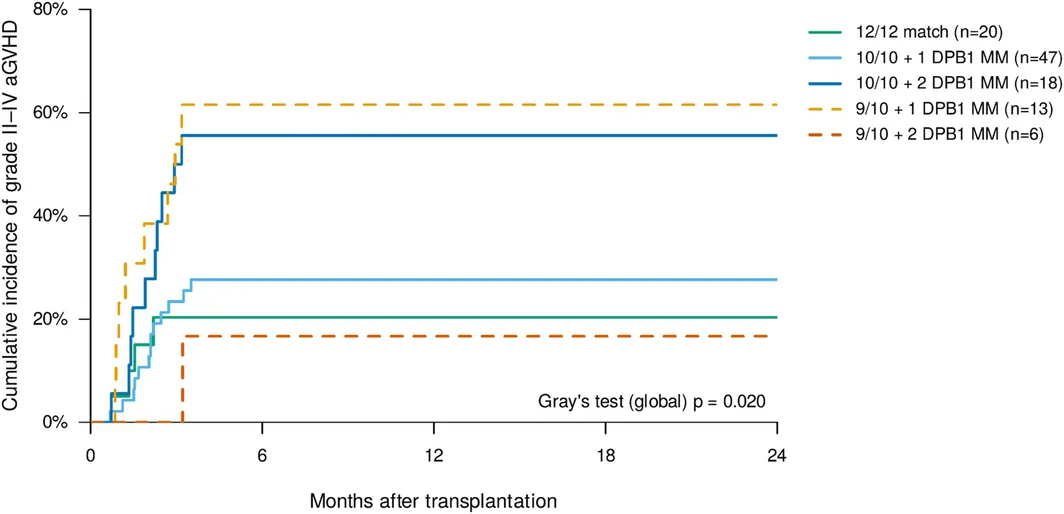
Cumulative incidence of Grade II–IV aGVHD by classical HLA match status and number of HLA‐DPB1 mismatches.

### 3.3 Covariate Selection for Multivariable Models

Six clinical variables (transplant period, disease risk, conditioning regimen, graft source, recipient CMV serostatus and GVHD prophylaxis) were screened in univariable Fine‐Grey models for Grade II–IV aGVHD among the 104 pairs with complete HLA‐DPB1 typing, using a pre‐specified threshold of *p* ≤ 0.20 (Table S1). Only disease risk crossed the pre‐specified threshold for consideration in the multivariable model (*p* = 0.18), whereas all other covariates showed no evidence of association. Disease risk was therefore retained as the adjustment variable in all subsequent multivariable Fine‐Grey models.

### 3.4 HLA‐DPB1 Match and Expression Models

A risk analysis of Grade II–IV aGVHD according to HLA‐DPB1 match in unrelated allo‐HCT recipients was performed (Table 2).

**TABLE 2:** Risk analysis of Grade II–IV aGVHD according to HLA‐DPB1 match in unrelated allo‐HCT recipients.

Risk analysis of HLA‐DPB1 mismatch	GvHD				Overall		Univariate			Multivariate		
	0‐I		II‐IV									
	*N*	%	*N*	%	*N*	%	sHR	CI (95%)	*p*	sHR	CI (95%)	*p*
Overall	67	64.4	37	35.6	104	100.0						
Match 12/12	16	23.9	4	10.8	20	19.2	reference			reference		
10/10 + 1 MM	34	50.7	13	35.1	47	45.2	1.33	0.42–4.18	0.630	1.56	0.53–4.57	0.420
10/10 + 2 MM	8	11.9	10	27.0	18	17.3	3.23	0.99–10.60	0.053	3.49	1.13–10.80	0.030
9/10 + 1 MM	5	7.5	8	21.6	13	12.5	3.52	1.01–12.3	0.049	4.46	1.45–13.70	0.009
9/10 + 2 MM	4	6.0	2	5.4	6	5.8	1.38	0.25–7.55	0.710	1.53	0.29–8.09	0.620
Disease Risk												
Intermediate/low	37	55.2	16	43.2	53	51.0	reference			reference		
high/very high	30	44.8	21	56.8	51	49.0	1.56	0.81–3.00	0.180	1.44	0.74–2.81	0.290

*Note:* Fine‐Grey subdistribution hazard model for Grade II–IV aGVHD (death without Grade II–IV aGVHD treated as competing risk), adjusted for Disease Risk. *n* = 104 donor‐recipient pairs with complete HLA‐DPB1 typing.

Abbreviations: CI, confidence interval; MM, mismatch; sHR, subdistribution hazard ratio.

Fine‐Grey competing‐risk regression suggested that HLA‐DPB1 mismatch category may be associated with increased risk of Grade II–IV aGVHD after adjustment for Disease Risk (Table 2). Compared with 12/12 HLA‐matched pairs, both 10/10 + 2 HLA‐DPB1 mismatches (sHR 3.49, 95% CI: 1.13–10.80, *p* = 0.030) and 9/10 + 1 HLA‐DPB1 mismatch (sHR 4.46, 95% CI: 1.45–13.70, *p* = 0.009) were associated with higher point estimates and 95% confidence intervals excluding 1 in the multivariate model, although the width of these intervals reflects the limited size of these subgroups and warrants cautious interpretation.

A descriptive analysis of concomitant mismatches at other HLA loci among 9/10 pairs found that DRB1 mismatch was present in 4 of 13 (31%) patients within the 9/10 + 1 HLA‐DPB1 mismatch group, versus 0 of 6 DRB1 within the 9/10 + 2 HLA‐DPB1 mismatch group, while HLA‐A locus mismatch was numerically more frequent in the 9/10 + 2 group (3/7, 43%) than in the 9/10 + 1 group (1/13, 8%). Given the very small subgroup sizes, these frequencies could not be statistically compared and are reported descriptively, without implying a causal or confirmed association.

When GVH‐direction mismatches were further stratified by predicted expression level of the mismatched allele (Table 3), the 10/10 + 2 mismatch category showed an increased risk of Grade II–IV aGVHD regardless of expression level (sHR 2.82, 95% CI: 1.07–7.43; *p* = 0.036). Among 9/10 + 1 mismatch pairs, an increased risk was suggested only for the high‐expression subgroup (sHR 3.33, 95% CI: 1.16–9.54; *p* = 0.025), although this estimate was based on only seven pairs and should be interpreted with caution.

**TABLE 3:** Risk analysis of Grade II–IV aGVHD according to HLA‐DPB1 GVH‐direction mismatch expression in unrelated allo‐HCT recipients.

Risk analysis of HLA‐DPB1 GVH mismatch expression	GvHD				Overall		Univariate			Multivariate		
	0–I		II–IV									
	*N*	%	*N*	%	*N*	%	sHR	CI (95%)	*p*	sHR	CI (95%)	*p*
Overall	67	64.4	37	35.6	104	100.0						
GVH Match expression	19	28.4	7	18.9	26	25.0	reference			reference		
10/10 + 1 MM low	15	22.4	8	21.6	23	22.1	1.42	0.50–4.03	0.510	1.51	0.56–4.02	0.410
10/10 + 1 MM high	16	23.9	3	8.1	19	18.3	0.79	0.22–2.86	0.720	0.96	0.28–3.34	0.950
10/10 + 2 MM	8	9.5	10	27.0	18	17.3	2.71	0.99–7.42	0.053	2.82	1.07–7.43	0.036
9/10 + 1 MM low	2	3.0	2	5.4	4	3.8	2.40	0.49–11.70	0.280	3.51	0.66–18.90	0.140
9/10 + 1 MM high	3	4.5	4	10.8	7	6.7	3.08	0.83–11.40	0.092	3.33	1.16–9.54	0.025
9/10 + 2 MM	4	6.0	3	8.1	7	6.7	1.15	0.24–5.65	0.860	1.25	0.26–6.03	0.780
Disease Risk												
Intermediate/low	37	55.2	16	43.2	53	51.0	reference			reference		
high/very high	30	44.8	21	56.8	51	49.0	1.56	0.81–3.00	0.180	1.36	0.69–2.66	0.380

*Note:* Mismatches classified by predicted expression level of the GVH‐direction mismatched allele (low vs. high).

Abbreviations: CI, confidence interval; MM, mismatch; sHR, subdistribution hazard ratio.

### 3.5 HLA‐DPB1 TCE Group and Directional Permissiveness Models

To characterise the association between HLA‐DPB1 mismatching and aGVHD Grade II–IV, we performed a competing‐risks regression analysis, first stratified by the T‐cell epitope (TCE) severity model (Table 4) and then by the directional permissiveness model (Table 5). In this cohort, all TCE‐1 and TCE‐2 mismatches were non‐permissive, whereas the permissiveness of TCE‐3 mismatches (core/non‐core) depended on the specific patient/donor allele combination.

**TABLE 4:** Risk analysis of Grade II–IV acute graft‐versus‐host disease (aGVHD) according to HLA‐DPB1 match and TCE model in unrelated allo‐HCT recipients.

Risk analysis of HLA‐DPB1 TCE group	GvHD				Overall		Univariate			Multivariate		
	0–I		II–IV									
	*N*	%	*N*	%	*N*	%	sHR	CI (95%)	*p*	sHR	CI (95%)	*p*
Overall	67	64.4	37	35.6	104	100.0						
Match 12/12	16	23.9	4	10.8	20	19.2	reference			reference		
10/10 TCE 1	5	7.5	8	21.6	13	12.5	3.52	1.06–11.70	0.040	3.27	1.05–10.20	0.041
10/10 TCE 2	13	19.4	7	18.9	20	19.2	1.80	0.51–6.33	0.360	2.67	0.73–9.71	0.140
10/10 TCE 3‐Core	10	14.9	4	10.8	14	13.5	1.30	0.33–5.05	0.710	1.51	0.41–5.58	0.540
10/10 TCE 3‐nonCore	14	20.9	4	10.8	18	17.3	1.10	0.26–4.68	0.900	1.13	0.29–4.40	0.850
9/10 TCE 1	1	1.5	3	8.1	4	3.8	6.04	1.27–29.90	0.028	3.64	1.28–10.30	0.015
9/10 TCE 2	2	3.0	3	8.1	5	4.8	3.65	0.81–16.40	0.091	5.26	1.12–24.70	0.035
9/10 TCE 3‐Core	3	4.5	1	2.7	4	3.8	1.07	0.14–7.97	0.950	1.57	0.16–14.90	0.700
9/10 TCE 3‐nonCore	3	4.5	3	8.1	6	5.8	1.69	0.30–9.47	0.550	2.32	0.40–13.50	0.350
Disease Risk												
Intermediate/low	37	55.2	16	43.2	53	51.0	reference			reference		
high/very high	30	44.8	21	56.8	51	49.0	1.56	0.81–3.00	0.180	1.39	0.61–3.14	0.430

*Note:* TCE groups reflect increasing predicted immunogenicity from TCE3 to TCE1 (TCE1 > TCE2 > TCE3); within TCE3, mismatches were further subclassified as core or non‐core (lower predicted immunogenicity for core vs. non‐core).

Abbreviations: CI, confidence interval; sHR, subdistribution hazard ratio.

**TABLE 5:** Risk analysis of Grade II–IV aGVHD according to HLA‐DPB1 match and permissiveness in unrelated allo‐HCT recipients.

Risk analysis of HLA‐DPB1 permissiveness	GvHD				Overall		Univariate			Multivariate		
	0–I		II–IV									
	*N*	%	*N*	%	*N*	%	sHR	CI (95%)	*p*	sHR	CI (95%)	*p*
Overall	67	64.4	37	35.6	104	100.0						
Match 12/12	16	23.9	4	10.8	20	19.2	reference			reference		
10/10 Permissive/core	10	14.9	4	10.8	14	13.5	1.30	0.33–5.04	0.710	1.53	0.41–5.68	0.530
10/10 Non‐permissive/non‐core—GvH	13	19.4	10	27.0	23	22.1	2.36	0.72–7.75	0.160	2.78	0.93–8.28	0.067
10/10 Non‐permissive/non‐core—HvG	12	12.6	6	16.2	18	17.3	1.66	0.46–5.95	0.440	2.14	0.61–7.48	0.230
10/10 Non‐permissive/non‐core—BID	7	10.4	3	8.1	10	9.6	1.53	0.33–7.17	0.590	1.38	0.30–6.35	0.680
9/10 permissive/core	3	4.5	1	2.7	4	3.8	1.07	0.14–7.96	0.950	1.59	0.17–15.10	0.690
9/10 Non‐permissive/non‐core—GvH	4	6.0	5	13.5	9	8.7	2.38	0.59–9.64	0.220	2.56	0.64–10.20	0.180
9/10 Non‐permissive/non‐core—HvG	2	3.0	4	10.8	6	5.8	4.84	1.12–20.9	0.035	5.68	2.06–15.70	0.001
Disease Risk												
Intermediate/low	37	55.2	16	43.2	53	51.0	reference			reference		
high/very high	30	44.8	21	56.8	51	49.0	1.56	0.81–3.00	0.180	1.50	0.75–2.98	0.250

*Note:* Permissive/core mismatches (TCE3‐core) reflect lower predicted immunogenicity; non‐permissive/non‐core mismatches were further subclassified by direction (GvH, HvG, or bidirectional [BID]).

Abbreviations: CI, confidence interval; sHR, subdistribution hazard ratio.

Predicted immunogenicity analyses of the mismatched TCE group suggested in the 10/10 pairs HLA‐DPB1 mismatch TCE1 may be associated with increased risk of Grade II–IV aGVHD after adjustment for Disease Risk (sHR 3.27, 95% CI: 1.05–10.20, *p* = 0.041), whereas in the 9/10 pairs, TCE1 and TCE2 categories suggested higher risk relative to the matched reference (sHR 3.64, *p* = 0.015 and sHR 5.26, *p* = 0.035; respectively), reaching statistical significance despite very small subgroup sizes (Table 4).

When the 104 pairs were reclassified according to the directional permissiveness model, non‐permissive/non‐core mismatches were further stratified by vector into graft‐versus‐host (GvH), host‐versus‐graft (HvG) and bidirectional (BID) mismatches and compared with permissive/core pairs and the HLA‐matched reference (Table 5). Among 10/10 pairs, GvH‐directed non‐permissive mismatches suggested an increased risk, though this did not reach statistical significance (sHR 2.78, 95% CI: 0.93–8.28; *p* = 0.067). Conversely, HvG‐directed mismatches suggested an increased risk of Grade II–IV aGVHD within the 9/10 pairs (sHR 5.68, 95% CI: 2.06–15.70; *p* = 0.001); however, this finding was based on a small subgroup (*n* = 6) and is not readily explained by the expected biology of HvG‐directed mismatches and should therefore be interpreted with caution.

### 3.6 Predicted Indirectly Recognisable HLA Epitopes (PIRCHE) Analyses

To evaluate whether the HLA mismatches identified in this cohort were associated with the risk of developing aGVHD through indirect T‐cell allorecognition, we performed a PIRCHE analysis across both the 10/10 and 9/10 HLA match groups. As expected, PIRCHE scores were considerably higher in 9/10 than in 10/10 pairs (PIRCHE‐I: median 8 [range 0–27] vs. 2 [0–9]; PIRCHE‐II: median 13 [6, 7, 8, 9, 10, 11, 12, 13, 14, 15, 16, 17, 18, 19, 20, 21, 22, 23, 24, 25, 26, 27, 28, 29] vs. 4 [0–19]; both *p* < 0.001), reflecting the additional HLA mismatch. PIRCHE‐I and PIRCHE‐II scores were therefore categorised into tertiles separately within each group. Because tertile boundaries were calculated independently, the score ranges defining each tertile are not directly comparable between groups and should be interpreted only within their respective group.

Among 10/10 HLA‐matched allo‐HCT recipients, the highest PIRCHE‐I tertile (scores 4.00–9.00), in which half of the pairs developed Grade II–IV aGVHD, showed a borderline increase in risk (sHR 3.36, 95% CI: 0.99–11.37; *p* = 0.052), whereas the two lower tertiles did not differ clearly from the reference (Table 6).

**TABLE 6:** Competing‐risk analysis of Grade II–IV aGVHD according to PIRCHE‐I mismatch score tertiles, stratified by degree of HLA matching (10/10 and 9/10) in unrelated allo‐HCT recipients.

Risk analysis of mismatch PIRCHE‐I (score)	GvHD				Overall		Univariate					
	0–I		II–IV							Multivariate		
	*N*	%	*N*	%	*N*	%	sHR	CI (95%)	*p*	sHR	CI (95%)	*p*
Overall	67	64.4	37	35.6	104	100.0						
HLA match 12/12	16	23.9	4	10.8	20	19.2	reference			reference		
10/10 (0.00–1.00)	19	28.4	8	21.6	27	26.0	1.40	0.42–4.73	0.586	1.40	0.42–4.69	0.590
10/10 (2.00–3.00)	14	20.9	6	16.2	20	19.2	1.44	0.40–5.18	0.578	1.36	0.38–4.91	0.635
10/10 (4.00–9.00)	9	13.4	9	24.3	18	17.3	2.97	0.87–10.11	0.081	3.36	0.99–11.37	0.052
9/10 (0.00–5.00)	3	4.5	4	10.8	7	6.7	3.20	0.82–12.51	0.094	3.95	0.92–16.96	0.065
9/10 (6.00–8.00)	5	7.5	1	2.7	6	5.8	0.82	0.08–8.51	0.866	0.60	0.06–6.45	0.677
9/10 (9.00–27.00)	1	1.5	5	13.5	6	5.8	6.38	1.74–23.42	0.005	7.02	1.93–25.52	0.003
Disease Risk												
Intermediate/low	37	55.2	16	43.2	53	51.0	reference			reference		
high/very high	30	44.8	21	56.8	51	49.0	1.56	0.81–3.00	0.180	1.79	0.91–3.94	0.147

*Note:* Tertile boundaries were calculated independently within each HLA‐match stratum (10/10 and 9/10); score ranges are therefore not directly comparable between strata.

Abbreviations: CI, confidence interval; sHR, subdistribution hazard ratio.

Among 9/10 pairs, the highest PIRCHE‐I tertile (Scores 10–27) had a higher estimated risk (sHR 7.02, 95% CI: 1.93–25.52; *p* = 0.003); however, this estimate was based on six pairs, five of whom developed Grade II–IV aGVHD (Table 6).

PIRCHE‐II tertiles and the risk of Grade II–IV aGVHD are shown in Table 7. Among 10/10 pairs, none of the PIRCHE‐II tertiles showed a clear increase in risk compared with the HLA 12/12 reference. Among 9/10 pairs all four pairs in the highest tertile (scores 20–29) developed Grade II–IV aGVHD. The resulting estimate (sHR 10.32, 95% CI: 2.50–42.61; *p* = 0.001) reflects near‐complete separation in a very small group and should be regarded as unstable.

**TABLE 7:** Competing‐risk analysis of Grade II–IV aGVHD according to PIRCHE‐II mismatch score tertiles, stratified by degree of HLA matching (10/10 and 9/10) in unrelated allo‐HCT recipients.

Risk analysis of HLA mismatch PIRCHE‐II (score)	GvHD				Overall		Univariate					
	0–I		II–IV							Multivariate		
	*N*	%	*N*	%	*N*	%	sHR	CI (95%)	*p*	sHR	CI (95%)	*p*
Overall	67	64.4	37	35.6	104	100.0						
HLA match 12/12	16	23.9	4	10.8	20	19.2	reference			reference		
10/10 (0.00–3.00)	17	25.4	9	24.3	26	25.0	1.72	0.52–5.72	0.376	1.61	0.48–5.43	0.443
10/10 (4.00–6.00)	12	17.9	5	13.5	17	16.3	1.43	0.38–5.45	0.596	1.53	0.40–5.80	0.530
10/10 (7.00–19.00)	13	19.4	9	24.3	22	21.2	2.15	0.64–7.17	0.215	2.24	0.68–7.40	0.185
9/10 (6.00–10.00)	4	6.0	4	10.8	8	7.7	2.69	0.68–10.61	0.159	2.83	0.70–11.52	0.145
9/10 (13.00–19.00)	5	7.5	2	5.4	7	6.7	1.42	0.25–8.04	0.692	1.31	0.22–7.85	0.765
9/10 (20.00–29.00)	0	0.0	4	10.8	4	3.8	11.24	2.88–43.85	< 0.001	10.32	2.50–42.61	0.001
Disease Risk												
Intermediate/low	37	55.2	16	43.2	53	51.0	reference			reference		
high/very high	30	44.8	21	56.8	51	49.0	1.56	0.81–3.00	0.180	1.42	0.72–2.80	0.306

*Note:* Tertile boundaries were calculated independently within each HLA‐match 10/10 and 9/10 groups; score ranges are therefore not directly comparable between groups.

Abbreviations: CI, confidence interval; sHR, subdistribution hazard ratio.

In the 9/10 group, the additional non‐DPB1 mismatch might have added its own epitopes to the PIRCHE‐DPB1 count either, since it is associated with markedly higher DPB1‐derived PIRCHE‐I (mean 9.3 vs. 2.7, *p* < 0.0001) and PIRCHE‐II (mean 15.7 vs. 6.4, *p* < 0.0001) scores compared with patients mismatched only at DPB1. Among the individual loci contributing to the 9/10 mismatch, HLA‐A mismatches showed the strongest association with higher PIRCHE‐I/II scores, though subgroup sizes for B, C and DQB1 were too small for definitive conclusions; no DRB1 mismatches occurred in this cohort. The number of mismatched DPB1 alleles (1 vs. 2) was not itself associated with higher scores.

### 3.7 Overall Survival Analysis

Overall survival analyses were performed for all 137 recipients over a 24‐month follow‐up period (Figure 3). In univariable log‐rank analyses (Table S2), transplant type for HLA match status (*p* = 0.205), transplant period (*p* = 0.007), disease risk (*p* = 0.086), recipient CMV serostatus (*p* = 0.043), donor age groups (*p* = 0.096) and donor/recipient gender combination (*p* = 0.087) met the pre‐specified threshold of *p* ≤ 0.20, whereas recipient age groups were not associated with overall survival (*p* = 0.425).

**FIGURE 3:**
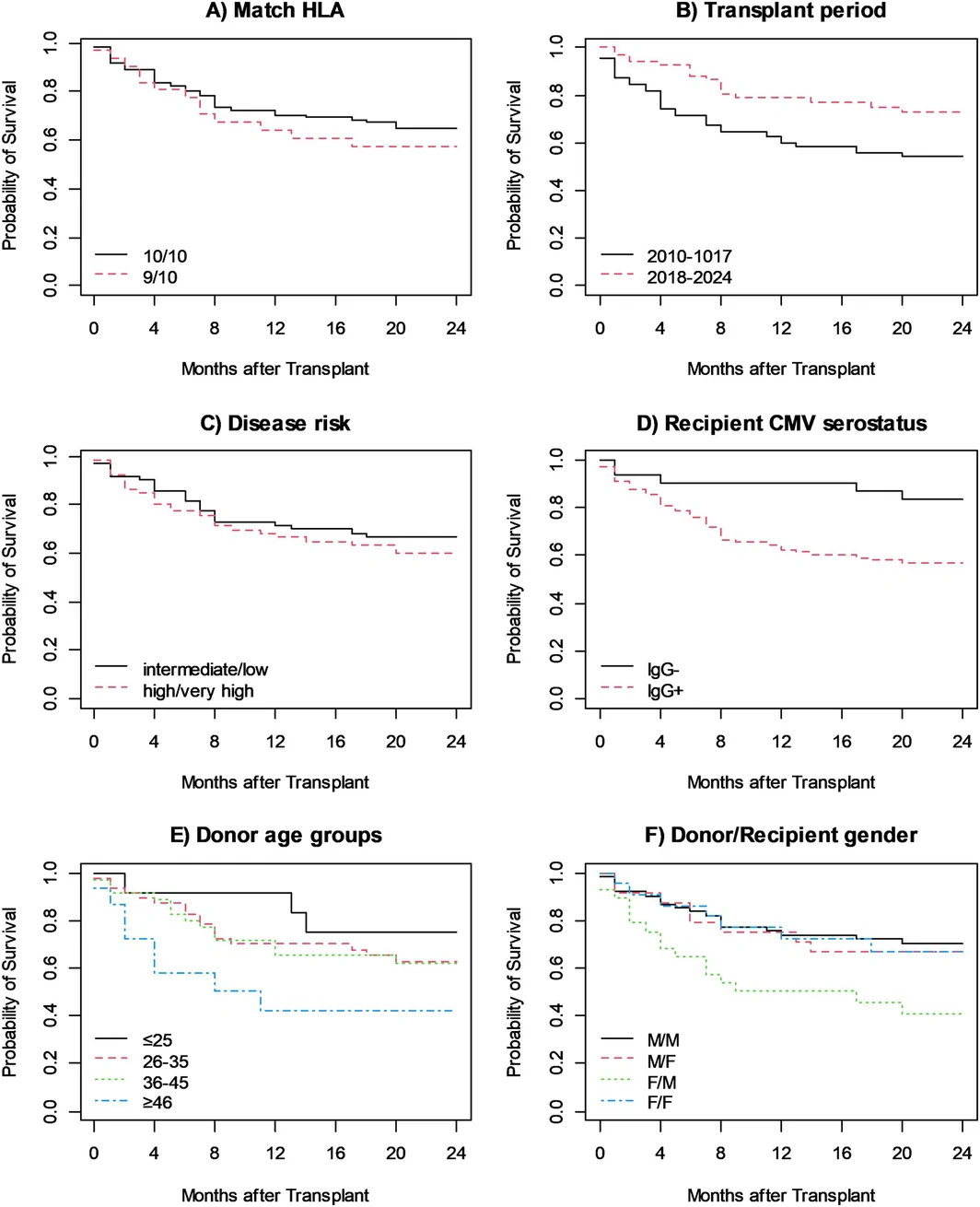
Overall survival curves of recipients undergoing allogeneic HCT stratified by transplant characteristics. Kaplan–Meier overall survival curves of 137 recipients who underwent unrelated allo‐HCT according to transplant characteristics. Survival analyses were stratified according to: (A) Match HLA (10/10 vs. 9/10); (B) transplant period (2010‐2017 vs. 2018‐2024); (C) Disease risk; (D) recipient CMV serostatus; (E) donor age groups and (F) donor/recipient pairs gender.

Among the 104 pairs with complete HLA‐DPB1 typing (43 deaths), none of the HLA‐DPB1 permissiveness models or PIRCHE categories was associated with overall survival, either in log‐rank analyses or in Cox models adjusted (Table 8) for transplant period, disease risk and recipient CMV serostatus (all *p* ≥ 0.20; Table S3).

**TABLE 8:** Multivariable Cox model for OS (*n* = 137, 64 deaths).

Covariate	HR	95% CI	*p*
HLA match: 9/10 versus 10/10	1.41	0.81–2.46	0.226
Transplant period: 20 10‐2017 versus 2018–2024	0.48	0.28–0.82	0.007
Disease risk: hig/very high versus intermediate/low	1.63	0.99–2.70	0.056
Recipient CMV serostatus: IgG+ versus IgG—	2.14	1.10–4.16	0.025
Donor/recipient pairs gender	1.63	0.99–2.69	0.057
Donor age groups (quartiles)	2.17	0.93–5.08	0.074

*Note:* Global model: likelihood‐ratio *p* = 0.001; concordance = 0.647. The proportional‐hazards assumption was satisfied for every covariate and globally (Schoenfeld global *p* = 0.26; per‐covariate p ranging 0.11–0.42). Patients transplanted in the more recent period (2018–2024) had significantly better survival, and recipient CMV IgG positivity was associated with significantly worse survival.

Relapse was also a covariate associated with death (35/64, 55%). Relapse‐related death occurred in 10/50 (20.0%) patients with Grade II–IV aGVHD and in 25/87 (28.7%) patients with Grade 0–I aGVHD (*p* = 0.31). Among deaths with known cause, relapses accounted for 40% (10/25) and 64% (25/39) of deaths, respectively (*p* = 0.075), a pattern that suggests a graft‐versus‐leukaemia effect accompanying aGVHD. Relapse‐related death did not differ appreciably by HLA‐DPB1 TCE permissiveness (match/permissive 15.8% vs. non‐permissive 25.8%, *p* = 0.33; among known‐cause deaths, 43% vs. 59%, *p* = 0.52).

## 4 Discussion

The present study evaluated the clinical impact of HLA‐DPB1 permissiveness using TCE, expression and PIRCHE models in unrelated donor allo‐HCT. Our findings demonstrated that HLA‐DPB1 incompatibilities, particularly non‐permissive mismatches and elevated PIRCHE scores, were associated with an increased risk of Grade II–IV aGVHD in both HLA 10/10 and HLA 9/10 transplant settings.

Among the 85 donor–recipient pairs initially matched at 10/10 HLA, only 20 (23.5%) were also matched at HLA‐DPB1; that is, 76.5% carried at least one HLA‐DPB1 mismatch. This proportion is in line with other groups of 10/10‐matched unrelated donor transplants, in which HLA‐DPB1 mismatches range from approximately 70% in an EBMT registry analysis [22] and in an earlier UK group [23] to more than 80% in other reports [19, 21]. The frequency of HLA‐DPB1 matching has been increasing over time, from 15% before 2011 to 31% in 2021–2022 [22]. This high mismatch rate is explained by the weak linkage disequilibrium between HLA‐DPB1 and the other HLA loci: HLA‐DPB1 lies at the centromeric end of the MHC and is separated from HLA‐DQB1 by at least one recombination hotspot [19, 20]. Mismatched HLA‐DP is therefore a hallmark of HLA‐matched unrelated donor transplantation, in contrast to HLA‐matched sibling transplantation [19].

Importantly, recipients carrying two HLA‐DPB1 mismatches showed a significantly increased risk of acute GVHD. These findings reinforce previous evidence suggesting that cumulative HLA‐DPB1 mismatches may enhance alloimmune activation and contribute to post‐transplant complications [19, 23, 30].

The TCE model demonstrated a significant association between TCE‐1 mismatches and Grade II–IV acute GVHD. Because TCE‐1 alleles are considered highly immunogenic, these results support the concept that the degree of T‐cell epitope disparity influences alloimmune responses after transplantation. Similar associations between non‐permissive HLA‐DPB1 mismatches and acute GVHD have previously been reported in large multicentre studies [25, 26, 28], and in a single‐centre cohort of 10/10 matched pairs [21].

Because the direction of a mismatch determines which cell population can recognise the disparity, we also analysed GvH‐, HvG‐ and bidirectional non‐permissive/non‐core mismatches separately (Table 5). Among HLA 10/10 pairs, although lacking statistical significance (*p* = 0.067), a higher incidence of aGVHD was observed for GvH‐direction DPB1 mismatches. This is consistent with a registry analysis of 3281 8/8‐matched transplants, in which directionality did not improve risk stratification [27]. A German multicentre study, however, attributed the aGVHD effect mainly to the GvH direction [28]. The high estimate for HvG‐direction mismatches among HLA 9/10 pairs (sHR 5.68, 95% CI: 2.06–15.70) rests on six patients and may be related to other HLA alleles mismatches.

PIRCHE analyses indicated that the risk of Grade II–IV aGVHD increased with higher numbers of predicted indirectly recognisable HLA epitopes. Among 10/10 pairs, the highest PIRCHE‐I tertile showed a borderline association with Grade II–IV aGVHD, whereas no clear association was observed for PIRCHE‐II. Among 9/10 pairs, the highest tertiles of both scores had markedly higher estimated risks; however, these estimates were based on four to six pairs and should be interpreted with caution. These findings are consistent with a potential contribution of indirect allorecognition to aGVHD [31] and with previous studies proposing PIRCHE to refine the assessment of HLA mismatches beyond conventional allele‐level matching [29, 32, 33]. Nevertheless, the relative contribution of PIRCHE‐I and PIRCHE‐II has varied across studies, and larger cohorts are needed to define clinically useful thresholds [29, 31].

Although survival analyses did not demonstrate statistically significant differences for any of the HLA‐DPB1 permissiveness models, this finding should be interpreted considering the limited number of events in each subgroup. Large registry studies have reported higher mortality with non‐permissive HLA‐DPB1 mismatches [25, 26], and the higher incidence of Grade II–IV aGVHD observed in our non‐permissive group did not translate into lower survival. Survival appeared to be driven mainly by non‐HLA factors. The lower mortality observed in 2018–2024 is consistent with the progressive improvement in transplant outcomes over time [34]. Recipient CMV seropositivity has been consistently associated with inferior survival after allogeneic HSCT [35]; in our setting, where most recipients were seropositive (77%), the seronegative reference group was small, and this estimate should be interpreted with caution. The higher mortality in female‐to‐male pairs agrees with studies attributing this effect to alloreactivity against Y chromosome‐encoded minor histocompatibility antigens [36], although the association has not been confirmed in large unrelated donor registries [37]. Finally, high/very high disease risk showed a trend towards lower survival [38], in line with relapse being the leading cause of death, particularly beyond the first year after transplant.

Furthermore, recipients transplanted from older donors showed poorer survival outcomes, consistent with previous studies [39, 40], supporting the use of younger donors for allo‐HCT whenever feasible [24]. Ageing is associated with reduced bone marrow cellularity [41], and advanced donor age has also been linked to lower nucleated cell doses, particularly in unmanipulated marrow grafts from donors older than 45 years [39]. These findings highlight the ongoing challenge of defining the optimal upper age limit for allo‐HCT donors and determining which donor‐related variables should be prioritised during donor selection [24, 42]. It is important to emphasise that all recipients in our cohort received calcineurin inhibitor‐based prophylaxis. Although this strategy has been associated with improved overall survival following allo‐HCT, a higher incidence of aGVHD has been reported in unrelated donor allo‐HCT recipients receiving conventional prophylaxis [43].

Paediatric recipients (≤ 15 years) represented a substantial fraction of the cohort (37.2%; 34.9% of HLA 10/10 and 45.2% of HLA 9/10 transplants). Children generally experience lower incidences of aGVHD and differ from adults in underlying disease, graft source and conditioning, which may have influenced the magnitude of the associations observed. Although recipient age was not associated with overall survival in our cohort (*p* = 0.425), the limited sample size precluded separate analyses in paediatric and adult recipients, and our findings may not be directly extrapolable to either population.

Taken together, our findings support a risk‐stratified approach to HLA‐DPB1 mismatching rather than a binary matched/mismatched assessment. Recent EBMT consensus recommendations state that, among the proposed models of permissive HLA mismatching, only the TCE and expression models have been validated in independent unrelated donor cohorts, and that their associations with outcome were established mainly under non‐PTCy prophylaxis, with HLA‐DPB1 status apparently losing relevance under PTCy [44]. Because all recipients in our cohort received calcineurin inhibitor‐based prophylaxis, our results reflect precisely the setting in which HLA‐DPB1 stratification remains clinically meaningful. In this context, the association between non‐permissive TCE mismatches and Grade II–IV aGVHD, together with the independent contribution of non‐HLA factors such as donor age, suggests that donor selection in centres using conventional prophylaxis may benefit from integrating HLA‐DPB1 permissiveness with other donor characteristics, as proposed in contemporary donor selection algorithms [43, 44].

The present study has some limitations. First, its retrospective single‐centre design may limit generalisability. Second, the relatively limited sample size may have reduced the statistical power for subgroup analyses, particularly survival analyses stratified by permissiveness categories. Nevertheless, this study provides relevant data regarding HLA‐DPB1 permissiveness in a Brazilian population undergoing unrelated donor transplantation.

In conclusion, our findings support the incorporation of HLA‐DPB1 permissiveness analyses into donor selection strategies for unrelated allo‐HCT. The combined use of TCE, expression and PIRCHE models may improve immunological risk stratification and contribute to reducing aGVHD incidence and optimising transplant outcomes.

## 5 Conclusion

Our results suggest that HLA‐DPB1 mismatches may be associated with an increased risk of aGVHD following unrelated allo‐HCT, particularly in the presence of non‐permissive TCE mismatches. Exploratory analyses of HLA‐DPB1 expression and PIRCHE scores identified additional associations with aGVHD in specific subgroups; however, these findings should be interpreted cautiously given the small subgroup sizes and wide confidence intervals. Together, these findings suggest that TCE, expression and PIRCHE models may provide complementary information for HLA‐DPB1‐related risk stratification in HLA 10/10 and HLA 9/10 transplantation settings, but their clinical utility for donor selection remains to be established.

Further multicentre studies with larger cohorts are warranted to validate the clinical utility of these permissiveness models and their incorporation into routine donor selection algorithms.

## Funding

This work was supported by Fundação Carlos Chagas Filho de Amparo à Pesquisa do Estado do Rio de Janeiro, E‐26/111.224/2013. Ministério da Saúde.

## Conflicts of Interest

The authors declare no conflicts of interest.

## Supporting information


**Table S1:** Covariate selection for multivariable models.
**Table S2:** Covariate selection for overall survival analysis.
**Table S3:** Overall survival according to HLA‐DPB1 permissiveness models and PIRCHE categories: global tests.

## Data Availability

The data that support the findings of this study are available on request from the corresponding author. The data are not publicly available due to privacy or ethical restrictions.
